# Learning to Make Collective Decisions: The Impact of Confidence Escalation

**DOI:** 10.1371/journal.pone.0081195

**Published:** 2013-12-06

**Authors:** Ali Mahmoodi, Dan Bang, Majid Nili Ahmadabadi, Bahador Bahrami

**Affiliations:** 1 Cognitive Systems Lab, Control and Intelligent Processing Centre of Excellence, School of Electrical and Computer Engineering, College of Engineering, University of Tehran, Tehran, Iran; 2 School of Cognitive Science, Institute for Research in Fundamental Sciences (IPM), Tehran, Iran; 3 Department of Experimental Psychology, University of Oxford, Oxford, United Kingdom; 4 Calleva Research Centre for Evolution and Human Sciences, Magdalen College, Oxford, United Kingdom; 5 Interacting Minds Centre, Aarhus University, Aarhus, Denmark; 6 UCL Institute of Cognitive Neuroscience, University College London, London, United Kingdom; Arizona State University, United States of America

## Abstract

Little is known about how people *learn* to take into account others’ opinions in joint decisions. To address this question, we combined computational and empirical approaches. Human dyads made individual and joint visual perceptual decision and rated their confidence in those decisions (data previously published). We trained a reinforcement (temporal difference) learning agent to get the participants’ confidence level and learn to arrive at a dyadic decision by finding the policy that either maximized the accuracy of the model decisions or maximally conformed to the empirical dyadic decisions. When confidences were shared visually *without* verbal interaction, RL agents successfully captured social learning. When participants exchanged confidences visually *and* interacted verbally, no collective benefit was achieved and the model failed to predict the dyadic behaviour. Behaviourally, dyad members’ confidence increased progressively and verbal interaction accelerated this escalation. The success of the model in drawing collective benefit from dyad members was inversely related to confidence escalation rate. The findings show an automated learning agent can, in principle, combine individual opinions and achieve collective benefit but the same agent cannot discount the escalation suggesting that one cognitive component of collective decision making in human may involve discounting of overconfidence arising from interactions.

## Introduction

The exchange of information between members of a group has been crucial to the success of the human species [1], [2]. However, surprisingly little is known about how we *learn* to integrate each other’s opinions when making decisions as part of a group [3]. To make effective group decisions, we must continuously evaluate the reliability of each other’s opinions and, perhaps more importantly, share and calibrate these subjective estimates in order to decide whose opinion is more likely to benefit the group. This task is complicated by the fact that the very process of social interaction may bias the information upon which our individual opinions are based [4]–[6].

Collective decisions e.g. jury verdicts, medical diagnosis or financial investment, are often characterized by uncertain choice between known alternatives. Uncertainty-ridden collective decision making has been subject to theoretical [7]–[9] and more recently, empirical examination [10]–[12]. A much more extensive body of work in social psychology of collective decision making has focused on knowledge refinement: opinion sharing and social influence have been studied in the context of knowledge of numerical facts (e.g. historical milestones, “*In what year did the second world war start?*”; descriptive statistics on demographics, “*what proportion of population in Framingham, MA are under 15 years old*?”; predicting the outcome of future sporting events) [13], [14]. However, both of these previous lines of work have generally assumed *stationarity* for social decision making by (often explicitly) positing that the reliability of individual opinions and the strategy for combining them stay constant over time.

Recently, a number of learning models have been proposed for social learning in non-cooperative contexts. Hampton and colleagues used reinforcement learning (RL) to examine how we infer the hidden intentions of those working against us [15], [16] used RL to describe how we integrate social advice with subjective information [16]. Behrens and colleagues [17] developed a Bayesian model to explain how we discount social advice based on an advisor’s history of trustworthiness. In the artificial intelligence domain, Mirian and colleagues developed a continuous Bayesian RL model to learn fusion of experts’ probabilistic decisions [18]. However, the primary focus of these studies was on game-theoretic approaches; consequently, for these models conflict of interest and inference of hidden intentions are the primary computational/cognitive hurdles. This is a different domain from the case of uncertainty-ridden social collective decision making where communication and integration information about uncertainty is the primary computational task. In summary, despite their intuitive appeal, theoretical and empirical examinations of dynamic aspects achieving a benefit from cooperation are scarce.

A demonstration of social learning in the context of collective decision making was recently reported [19]. Dyad members participated in a visual perceptual experiment in which they estimated their confidence in their individual decisions about a visual stimulus on every trial, but were also required to make joint decisions whenever their individual decisions conflicted. The results indicated that dyadic performance changed over time. Dyads did not initially exceed their better member. But with time, groups accumulated a robust collective benefit. Critically, the results showed that dyad members’ communicated confidence ratings changed *relative to each other* over time. Such a demonstration of dynamic changes in social collective decision making mean that previous simpler models that assumed stationary dynamics [10], [11] must be complemented by more sophisticated models that could take into account such dynamics. To address this problem, we developed a model for social learning in collective decision making based on the principles of reinforcement learning [20], [21].

In addition, we used this modelling exercise to address another question raised earlier. Bahrami and colleagues in [19] showed that dyad members who first made an individual decision and then verbally discussed a joint decision outperformed dyad members who were also asked to explicitly rate their confidence in their individual decision Thus, explicit introspection and verbal communication interacted *sub-additively* in contributing to collective decision making. Interestingly, dyads who communicated only via explicit introspection (without verbal communication), did not do any better. As such, the question how engaging in different modes of expressing one’s confidence may interfere with one another remains open. We asked if combining verbal and visual confidence sharing affects the dynamical aspects of learning in social collective decision making. We used the empirical data from a previous study [19] to compare the success of our RL-based model in explaining dyadic behaviour and to identify the possible psychological mechanism that might have led to differences in collective benefit for various modes of communication.

## Methods

### The Experiment

The local ethics committee (The Interacting Mind Ethics Committee at Aarhus University) approved all experiments, and written informed consent was obtained from all participants. The stimuli parameters and the procedure have been described in detail elsewhere [19]. In brief, 58 healthy male adult participants (mean age ± std: 23.5±2.5) were paired into 29 dyads and participated in one of two conditions (14 dyads in a Visual condition and 15 dyads in a Verbal/Visual condition – see below). Members of each dyad knew each other beforehand. Each participant was only recruited for one of the two conditions.

**Figure 1:**
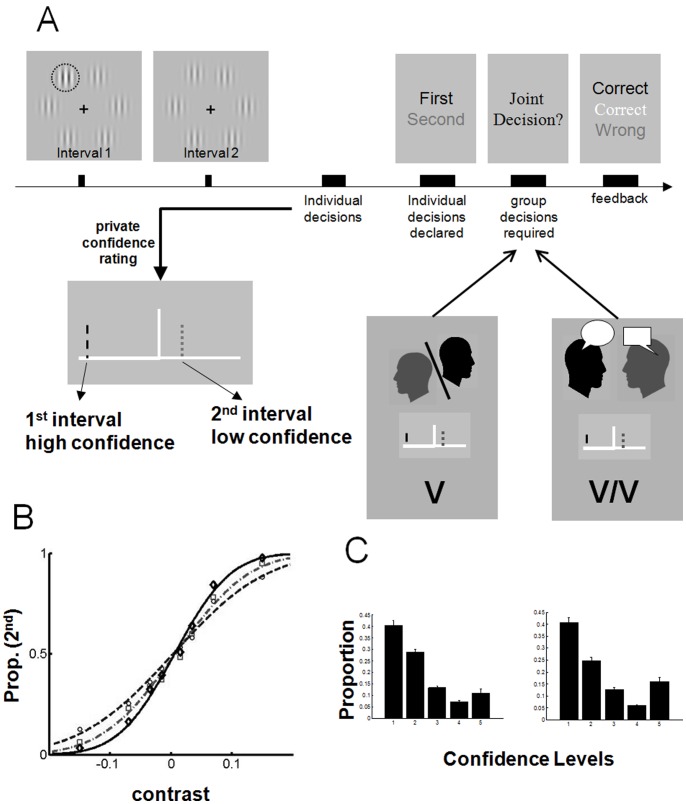
(A) Stimulus, experimental procedure and modes of communication. In each trial participants observed two consecutive stimulus intervals and then announced their private decisions about which interval contained the oddball (here illustrated by the dotted outline). Participants reported their confidence in private. Individual decisions were then announced, and in cases of disagreement participants saw each other’s confidence rating (in both conditions) and also talked to each other (only in V/V condition) in order to reach a joint decision. Feedback was provided at the end of each trial (B). The average psychometric function plots the proportion of trials in which the 2nd interval was chosen against the contrast difference between oddball and distractors. A highly sensitive observer would produce a steeply rising psychometric function with a large slope. Circles, performance of the less sensitive observer (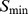) of the dyad; grey squares, performance of the more sensitive observer (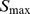); and black squares, performance of the dyad (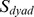). (C) Distribution of confidence levels in the Visual and Visual/Verbal conditions. Error bars are 1 SE.

In each trial, the dyad members first made an individual decision about a briefly presented visual stimulus (i.e. whether a target occurred in a first or second viewing interval) and indicated their confidence in this decision on a scale with 5 steps (Figure 1A). The individual responses (i.e. decision and confidence) were then publicly displayed for both dyad members. In the case of disagreement (i.e. the dyad members independently selected different intervals), the dyad members were required to make a joint decision. In the verbal/visual (V/V) condition, the dyad members had access to each other’s responses (i.e. decision and confidence) and were also allowed to talk to each other about what might be the right decision. In the visual (V) condition, the dyad members only had access to each other’s responses. In both conditions, for each disagreement trial, one of the two dyad members was randomly nominated to indicate the joint decision. On each trial, visual target’s contrast was randomly chosen from 4 values, spanning very easy (high contrast) to very difficult (low contrast) decisions. Each dyad completed 16 blocks of 16 trials, giving rise to 256 trials in total.

### Estimating the Individual and Collective Performance

For each decision maker (i.e. individuals and the dyad as a whole), a psychometric function was constructed by calculating the proportion of trials in which the target was reported seen in the second interval against the target contrast (i.e. Δ*c*, the target contrast in the second interval minus the target contrast in the first – see Figure 1B). The resulting curves were fit to a cumulative Gaussian function with parameters bias, *b*, and variance, σ^2^ using a probit regression model (*glmfit* function in Matlab, Math works Inc). A decision maker with bias *b* and variance σ^2^ would have a psychometric function *P*(Δ*c*) where Δ*c* is the target contrast difference, given by
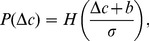
(1)


Where *H*(*z*) is the cumulative Normal function,
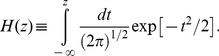
(2)


Given the above definitions for *P*(Δ*c*), we see that the decision variance is related to the maximum slope of the fitted psychometric curve at its point of inflection, denote *s*, via
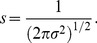
(3)


A steeply rising curve has a large slope, indicating small variance and thus high sensitivity to the target contrast. We used this measure to quantify the individuals’ and the dyad’s sensitivity. We defined *collective benefit* as the ratio of the dyad’s slope (*s_dyad_*) to that of the more sensitive dyad member (i.e. the dyad member with the steeper slope, *s_max_*); a value above 1 indicated that the dyad managed to obtain a benefit over and above its better observer.

### Modelling

We used reinforcement learning (RL) to construct a dynamic model of the dyadic choice behaviour. An RL agent searches for a behavioural policy that maximizes its expected reward. The RL agent solves this problem by estimating the expected reward –called *value*–of the possible *actions* for each *state* that the agent may encounter in its environment [20], [21]. In our case, each state(*s*) is identified by the pair of confidences (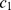 and 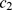) reported by the dyad members in each trial. The action 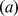 is the joint decision (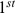 or 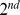 interval) adopted by the dyad. The reward 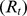 in trial *t* is +1 if the decision turns out to be correct and −1 otherwise. The behaviour policy adopted by the RL agent is the probability distribution that the agent assigns its two possible actions for each state. We used a single-step version of the Temporal Difference (TD) learning algorithm (Sutton, 1998). In this algorithm, trial-by-trial, the agent updates the value of the action-state pair (*s,a*) pertaining to that trial:
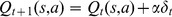
(4)where 0≤α≤1 is free learning rate parameter and 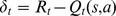 is the *prediction error.*

### Reduction of the State Space

In both conditions, (see above and Figure 1A) the individual confidence estimates took integer values from −5 (high confidence for first interval) to +5 (high confidence for second interval) excluding zero. Therefore, the two dimensional 10×10 state space 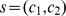 had100 *possible* combinations. This number of states was too large for the learning algorithm to handle and converge meaningfully considering that the total number of trials was 256. Moreover, we observed that participants’ used the higher confidence (4 & 5) levels much less frequently (see Figure 1 C). Therefore, we transformed the state space by collapsing the two highest levels of confidence (i.e. −/+4 and −/+5 were relabelled as −/+4). Given our models’ preference for smaller state-spaces, one may wonder whether empirical interpersonal communication might have been more successful if a sparser confidence space (e.g. with 3 rather 5 levels) was offered to the participants. Unfortunately, the behavioural results described here cannot tell us much about the human observers’ preferred resolution of confidence space. Future research in collective decision making could address such possible role of resolution of information. To ensure the generality of our findings, we also tried a number of similar transformations of the state space and our results were qualitatively replicated.

### Max Accuracy RL

For each dyad, we divided the experimental data into three time bins and for each time bin. We observed that people’s confidence reporting changes across time (see Escalation of Confidence). Previously, it was shown [19] that the mutual relationship between confidence ratings of dyad members changed across time. Bahrami et al [19] calculated the alignment of confidence across trials and found that the dynamics of the chance in this ratio was only observable when the data were split into three or more bins (See their Figure 8A in ref. [19]). One way to deal with such a non-stationary confidence reporting is to tune the α parameter (learning rate) every few trials. Instead, and to avoid model complexity we divided the data into three equal bins and restarted the learning process from the beginning in each bin. By doing so, we could cope with the previously observed non-stationary nature of confidence reporting. We also tried dividing the data into more bins, but number of trials in each bin wouldn’t be sufficient for the analysis. We tried modelling the entire time-series as one whole session (i.e. without restarting the learning by using one bin) as well. The model fitness to dyads’ slope was best with three bins. Nevertheless, the main findings were the qualitatively same for three and one bin analysis. We ran the learning algorithm with a fixed learning rate, the free parameter (0≤α≤1) in eq. (4). Within each bin, we searched for the learning rate that produced the maximum slope (defined in eq. 3). Then we computed the RL agent’s overall slope (see Table S1 for the pseudo code). Since we wanted this slope to be comparable to dyadic performance measures across the entire experiment, we collapsed the whole data of the three bins and calculated the slope of the whole trials. At the beginning of each run of learning algorithm for each subset, we initialized the Q-values to zero. The Q-values were updated using (eq. 4). In each trial the agent used a greedy policy for decision making:
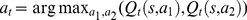
(5)


Where 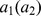 corresponded to 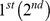 interval respectively. In the first occurrence of each state, where 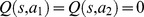, the agent took the action that had higher confidence; i.e.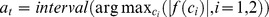 where *interval(l)* is the interval associated to the confidence level *l* and *f*(.) is the state definition function; see **Reduction of the state space**.

### Max Similarity RL

The accuracy maximizing RL treated each dyad as one functional unit. One may argue, however that in our experiments, even though every disagreement trial involves arbitration between dyad members, the joint decision was eventually made by the dyad member who was nominated to indicate the decision. As such, each dyad may better be described as a combination of *two* decision makers. In order to address this possibility, we fitted separate RL models to the joint decisions indicated by each dyad member, searching for the learning rate that most closely fitted the individual dyad member’s choice behaviour when responded on behalf of the dyad. All other model details were the same as those of the accuracy-maximizing RL model.

## Results

### Max Accuracy RL

**Figure 2:**
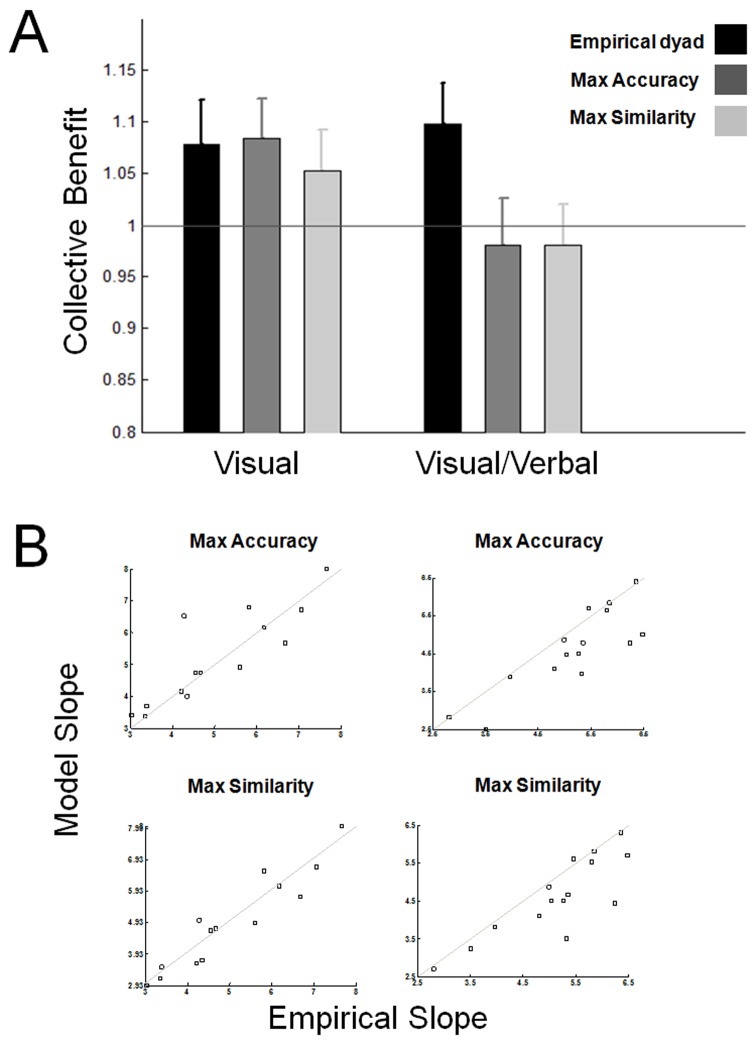
Comparison of empirical and modelling outcomes. (A) Average collective benefit (CB, *s_model_*/*s_max_*) is plotted for the empirical (black) data as well as the RL models (light and dark grey). In the visual condition, the RL model successfully accrued a significant collective benefit compared to the dyad’s best member’s sensitivity. Error bars are 1SE. (B) Scatter plots show the relation between model predictions and empirical data for Max Accuracy (top row) and Max Similarity (bottom row): both modelling approaches did a good job of case-by-case predicting the empirical dyadic slope in the Visual condition (left column). But in the Visual/Verbal condition (right column), the RL models were consistently inferior to empirical performance.

To compare the empirical dyadic decision with those of the RL agents, we computed the collective benefit (CB) obtained by the model (*s_model_*/*s_max_*, Figure 2A, dark grey bars)and compared it to empirical collective benefit obtained by the dyads (*s_dyad_*/*s_max_*, Figure 2 A, black bars)for the V and V/V conditions. In the V condition, the RL model successfully accrued a significant collective benefit compared to the dyad’s best member’s sensitivity (*t*(13) = 2.6; *p<*0.01; one sample t-test comparing logarithm mean CB to 0). To avoid heavy tale distribution, we applied the statistical tests on the log-transformed ratios. Furthermore, this collective benefit obtained by the model was comparable to that empirically achieved by the dyads. The upper left panel in Figure 2 B shows that the accuracy maximizing RL model did a good job of case-by-case predicting the empirical dyadic slope in the Visual condition. In the Visual/Verbal condition, however, the RL model did not achieve any significant collective benefit (*t*(14) = −.71; *p>*0.48; one sample t-test comparing logarithm mean CB to 0). Moreover, the collective benefit accrued by the RL model was significantly less than that achieved by the dyads (paired t-test comparing logarithm CB for model and the dyads; *t*(14) = −3.74; *p<*0.003; Figure 2A and 2B upper right panel). Finally, testing our main hypothesis directly revealed that the concordance between the RL model and empirical data (*s_model_*/*s_dyad_*) was significantly higher in the V compared to V/V conditions (independent sample t-test; *t*(27) = 2.3; *p<*0.04).

### Max Similarity RL

Here we modelled the dyadic decision making process as the combination of two parallel, concurrent reinforcement learning processes, one for each dyad member. We wanted to see if conceiving of the dyad as the aggregation of two separate decision makers rather than a singular unit (as in above) would enhance the RL model’s concordance with the empirical data. The aggregate RL agent conferred larger collective benefit in the V compared to V/V (independent t-test; t(27) = 2.1;p = 0.034). It was a also good predictor of dyadic performance in the V (paired t-test; t(13) = .24; p = 0.8; Figure 2B, lower left panel) but not in the V/V (paired t-test; t(14) = −3.5; p = 0.0035; Figure 2B, lower right panel) condition. In sum, these results did not show any qualitative difference between the dyad as an aggregate (Max Similarity) and dyad as a unit (Max Accuracy) modelling approaches. Therefore, through the rest of the paper we only focus on the simpler Max Accuracy RL model. However, caution must be exercised in direct comparison of these two approaches since they employ quite different details (e.g. number of free parameters).

The results suggested that availability of verbal communication affected the learning strategy employed to arrive at dyadic decisions. In the Visual condition, dyadic behaviour was consistent with the simple RL strategy encapsulated by eq. 4 and 5. However, in the Visual/Verbal condition, even though dyads achieved a comparable level of collective benefits, their behaviour was not consistent with the same RL strategy. What could the impact of verbal communication on collective decision making be that led to such divergent strategies in the V and V/V conditions?

One possibility is that direct, verbal interaction might have affected how the individuals express their shared confidence. In an elegant study, Shergill and colleagues [22] had participants engage in a tit-for-tat game of exchanging forces where two participants took turns at applying pressure (using their right index finger) to each other’s left index finger. Importantly, both participants were instructed to apply *the same* amount of pressure that their partner had applied to them in the preceding turn. Surprisingly, the applied force escalated rapidly even though instructions emphasized maintaining equality. Agents applied more and more force upon each other. In a second experiment, Shergill and colleagues [22] demonstrated that force escalation critically depends on *direct* interaction. When participants applied forces via an intermediary device – transforming a joy-stick movement to force –force escalation was substantially reduced.

We conjectured that direct interaction might have a similar effect on confidence judgements. Indeed, previous research suggests that making a decision as part of a group leads to increases in confidence that are not mirrored in accuracy [14]. Based on these findings, we hypothesised that direct interaction led to an escalation of decision confidence that was not mirrored in increased sensitivity (i.e. the slope of the psychometric function). Moreover, similar to escalation of forces, one may expect the boost in confidence to build up progressively over time. Finally, we predicted that if the failure of the Max Accuracy RL models to account for the collective decisions is due to confidence escalation, then the collective benefit achieved by the RL algorithm should be correlated with the speed of confidence escalation across dyads.

### Escalation of Confidence

There was no difference in individual participants’ slope between conditions (independent samples *t*-test; *t*(56) = 0.06, *p*>.94). However, mean absolute confidence expressed by participants (averaged over all trials) was significantly higher in the V/V compared to V condition (independent samples *t*-test; t(56) = −2.29, *p*<.03). These results corroborated the previous findings (Heath and Gonzalez,1995) that verbal interaction leads to increased confidence without improving accuracy.

**Figure 3:**
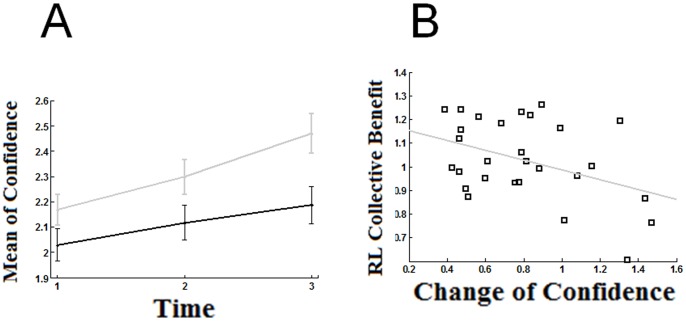
Confidence escalation and its correlation with collective benefit. (A) Mean absolute decision confidence (across participants) is plotted against time bins. Each time bin corresponds to one third of the trials. Black and grey lines refer to V and V/V conditions, respectively. Error bars are 1 SE. (B) Collective benefit obtained by the best fitting accuracy-maximizing RL model is plotted against change of confidence across the 3 time bins.

To assess the build up of confidence over time, we again divided the data into the 3 time bins devised and employed a 2 (V and V/V conditions) by 3 (time bins) ANOVA. The main effects of experimental condition and time were both significant (Figure 3 A; for condition, *F*(1,56) = 4.85, *p* = 0.03; for time *F*(2,112) = 26.71, *p*<0.001; Figure 3 B). The interaction between condition and time bin was nearly significant (*F*(,2,112) = 2.73, *p* = 0.070) lending support to the hypothesis that direct interaction accelerated the escalation of confidences. Direct comparison between conditions in each time bin showed no significant difference in confidence in the first time bin (*t*(56) = 1.56, *p*>0.12; independent samples t-test), a near-significant difference in confidence in the second time bin (independent t-test; *t*(56) = −1.89, *p* = 0.06) and a significant difference in confidence in the third time bin (independent t-test; *t*(56) = 2.6,*p*<.02). A similar 2 by 3 ANOVA on individual sensitivity showed no significant effects (*p*>.05).

We then tested the hypothesized relationship between speed of confidence escalation and failure of the RL model. Since this prediction was independent of the mode of communication, we tested the correlation after collapsing the data from the two conditions. We first quantified the change in mean absolute confidence from bin 1 to bin 3 for each individual by:
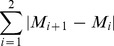
Where *M_i_* is the average absolute confidence of a participant in time bin *i*. Then for each dyad, we calculated the sum of this value from the constituting individuals. A negative correlation (Pearson *r* = −.405; *p*<.03; *R^2^* = −10.66) was found between the dyadic cumulative change in absolute confidence and the collective benefit obtained by the Max Accuracy RL model for each dyad.

## Discussion

We employed a reinforcement learning [20], [21] approach to develop a model for social learning in collective decision making via confidence sharing. We used the empirical data obtained from human participants in a previous work and trained two simple RL algorithms that, on a trial by trial basis, combined the participants’ expressed level of confidence to arrive at a dyadic decision. Learning involved finding the appropriate policy for mapping individual confidence pairs to dyad decisions that either maximized the accuracy of the model or most closely conformed to the dyadic decisions.

We found that both approaches were similarly successful at explaining the empirical findings in the Visual condition where dyad members shared their confidences through a graphical interface *without* interacting verbally with one another. This result helps us draw a clearer picture of how individuals combine their own uncertainty-ridden decision with those expressed by others. The simplicity of the learning algorithm, which essentially boils down to equations 4 and 5 (see Methods), is of great value in helping us form an idea about the mechanism of how the dyads may have learned from previous rounds of interaction towards arbitrating the current disagreement.

This finding also demonstrates that communication of introspection by Visual means alone is rich enough to ensure collective benefit even by an automated learning agent such as the RL models employed here. This is consistent with a recent study [12] which showed that pooling subjective confidences from multiple non-communicating observers leads to collective benefit. Both [12] and the current study focused on perceptual decisions, yet it is difficult to compare the quantitative magnitude of collective benefits delivered by each method. Applying the Maximum Confidence Slating (MCS) algorithm [12] to our data is problematic because in MCS, non-communicating observers are handpicked *post-hoc* by the experimenter to form “virtual” dyads according to the *similarity* of their individual performances. This is not the case for the current work and individuals comprising a dyad are fixed. Future research will be needed to clarify the possible differences between automated social learning algorithms (such as implemented here) and the *post-hoc* schemes that depend on an experimenter’s direct influence.

In the Visual/Verbal condition, on the other hand, where participants exchanged confidences visually *and* interacted verbally, the same RL models were unable to achieve any collective benefit and significantly deviated from predicting the dyadic behaviour. These diverging findings from the Visual versus Visual/Verbal conditions can help us infer the direction of interference between introspection and collective decision making. Bahrami and colleagues [19] showed that dyads achieve more collective benefit if they make their private decisions (Figure 1) *with* verbal communication but *without* explicit confidence rating. That finding suggested that introspection (i.e. explicit confidence rating) which is a cognitively demanding process [23], [24] may interfere with verbally mediated collective decision making. An open question was whether this interference is unidirectional or, rather, verbal interaction could also interfere with the process of introspection.

Meanwhile, previous works showed that verbal communication alone is also adequate for ensuring collective benefit [11], [25]. Since verbally and visually communicated confidences are, by definition, meant to convey the same information (i.e. the subjective probability of accurate decision) substantial redundancy must be shared between them. As such, the fact that the empirical benefits of the two channels did not add up to additional collective benefit in the Visual/Verbal condition (Figure 3A, compare black bars) may simply be a trivial consequence of such redundancy rather than any form of active interference.

The failure of the RL models in the Visual/Verbal condition rejects the redundancy alternative and presents strong evidence for the interference account. Some active form of *interference* between the two channels of communication renders the visually conveyed information much less informative about decision uncertainty: in the Visual/Verbal condition, the same RL models (with identical structural complexity and number of parameters to Visual condition)did not achieve any collective benefit from utilizing the visually shared confidence. Thus, our findings using computational modelling go beyond earlier work [19], [24] by clearly demonstrating the interfering impact of direct verbal interaction on the process of introspection and explicit confidence rating.

Our subsequent follow-up behavioural analysis showed that as participants went through the experiment, they grew progressively more confident in their decisions; this boost in confidence was much more pronounced with verbal communication (Figure 3A) and was inversely correlated with success of the RL model applied to confidence estimates (Figure 3B). These results help further clarify the nature of the interference between introspection and social interaction in the form of confidence escalation (Heath and Gonzalez, 1995; Shergill et al 2003).

An interesting aspect of our behavioural findings is that the collective benefit obtained by the dyads was not affected by the greater confidence escalation under V/V (vs. V) condition (Figure 2A, black bars). This raises the possibility that participants in the V/V condition were simply ignoring the confidence ratings and focused on the verbal communication. This account would require that collective benefit in the V/V condition be as good as when participants communicate exclusively verbally without any explicit confidence rating. Bahrami et al in [19] showed that collective benefit is significantly larger under verbal-only (versus V/V) communication ruling out the possibility of ignoring the confidence ratings in the V/V condition. Shergill et al in [22] argued that human agents engaged in force escalation underestimate the force they apply to their partner because they implicitly discount their own applied force. It is likely that here too, in V/V condition agents have some implicit understanding of the escalating nature of their shared confidences which may help them discount the trend and achieve empirical collective benefits comparable to that obtained in the Visual condition where confidence escalation is much less pronounced. Such implicit understanding of the underlying dynamics, however, is not available to the RL model leading to its failure in the Visual/Verbal condition. An important question for future research would be whether agents are indeed aware of such trends or not and if they could learn to minimize their interfering impact on communication towards collective benefit.

## Supporting Information

Table S1 **Pseudocode for RL algorithm.** (A) Maximum accuracy and (B) maximum similarity. In maximum accuracy (maximum similarity) for each dyad (individual) we first transformed the confidence ratings (see Methods) and then ran the learning algorithm with a fixed learning rate for each subset of the experimental data. We searched for the learning rate that maximized the slope (trial by trial similarity of model and individual) over each three subsets of the trials; then for each trial, we assigned decisions to dyads based on the winning learning rate model and finally calculated the overall dyadic slope for each dyad.
(DOCX)

## References

[1] Bowles S, Gintis H (2011) *A cooperative species: Human reciprocity and its evolution*. Princeton University Press.

[2] Richerson PJ, Boyd R (2008) *Not by genes alone: How culture transformed human evolution*. University of Chicago Press.

[3] Hastie R, Kameda T (2005) The robust beauty of majority rules in group decisions. Psychol. Rev., vol. 112, no. 2, p. 494. 10.1037/0033-295X.112.2.494 15783295

[4] Zajonc RB (1965) *Social facilitation*. Research Center for Group Dynamics, Institute for Social Research, University of Michigan.

[5] Asch SE (1951) Effects of group pressure upon the modification and distortion of judgments. Groups Leadersh. Men S, 222–236.

[6] Bahrami B, Didino D, Frith CD, Butterworth B, Rees G (2013) Collective enumeration. J. Exp. Psychol. Hum. Percept. Perform., vol. 39, no. 2, 338–347. 10.1037/a0029717 PMC3607463 22889187

[7] Bovens L, Hartmann S (2004) Bayesian epistemology. OUP Cat.

[8] Nitsan S, Parousch J (1985) *Collective decision making: an economic outlook.* CUP Archive.

[9] Austen-Smith D, Banks JS (1996) Information aggregation, rationality, and the Condorcet jury theorem. Am. Polit. Sci. Rev., 34–45.

[10] Sorkin RD, Hays CJ, West R (2001) Signal-detection analysis of group decision making. Psychol. Rev., vol. 108, no. 1, p. 183. 10.1037/0033-295x.108.1.183 11212627

[11] Bahrami B, Olsen K, Latham PE, Roepstorff A, Rees G, et al.. (2010) Optimally interacting minds. Science, vol. 329, no. 5995, 1081–1085. 10.1126/science.1185718 PMC3371582 20798320

[12] Koriat A (2012) When Are Two Heads Better than One and Why?. Science, vol. 336, no. 6079, 360–362. 10.1126/science.1216549 22517862

[13] Yaniv I (2004) The benefit of additional opinions. Curr. Dir. Psychol. Sci., vol. 13, no. 2, 75–78.

[14] Heath C, Gonzalez R (1995) Interaction with others increases decision confidence but not decision quality: Evidence against information collection views of interactive decision making. Organ. Behav. Hum. Decis. Process., vol. 61, no. 3, 305–326.

[15] Hampton AN, Bossaerts P, O’Doherty JP (2008) Neural correlates of mentalizing-related computations during strategic interactions in humans. Proc. Natl. Acad. Sci., vol. 105, no. 18, 6741–6746. 10.1073/pnas.0711099105 PMC2373314 18427116

[16] Biele G, Rieskamp J, Krugel LK, Heekeren HR (2011) The neural basis of following advice. PLoS Biol., vol. 9, no. 6, p. e1001089. 10.1371/journal.pbio.1001089 PMC3119653 21713027

[17] Behrens TE, Hunt LT, Woolrich MW, Rushworth MF (2008) Associative learning of social value. Nature, vol. 456, no. 7219, 245–249. 10.1038/nature07538 PMC2605577 19005555

[18] Mirian MS, Ahmadabadi MN, Araabi BN, Siegwart RR (2011) Learning active fusion of multiple experts’ decisions: An attention-based approach. Neural Comput., vol. 23, no. 2, 558–591. 10.1162/NECO_a_00079 21105824

[19] Bahrami B, Olsen K, Bang D, Roepstorff A, Rees G, et al.. (2012) What failure in collective decision-making tells us about metacognition. Philos. Trans. R. Soc. B Biol. Sci., vol. 367, no. 1594, 1350–1365. 10.1098/rstb.2011.0420 PMC3318766 22492752

[20] Sutton RS, Barto AG (1998) *Reinforcement learning: An introduction.* vol. 1. Cambridge Univ Press.

[21] Szepesvári C (2010) Algorithms for reinforcement learning. Synth. Lect. Artif. Intell. Mach. Learn., vol. 4, no. 1, 1–103.

[22] Shergill SS, Bays PM, Frith CD, Wolpert DM (2003) Two eyes for an eye: the neuroscience of force escalation. Science, vol. 301, no. 5630, 187–187. 10.1126/science.1085327 12855800

[23] Ericsson KA, Simon HA (1980) Verbal reports as data. Psychol. Rev., vol. 87, no. 3, p. 215.

[24] Corallo G, Sackur J, Dehaene S, Sigman M (2008) Limits on Introspection Distorted Subjective Time During the Dual-Task Bottleneck. Psychol. Sci., vol. 19, no. 11, 1110–1117. 10.1111/j.1467-9280.2008.02211.x 19076482

[25] Fusaroli R, Bahrami B, Olsen K, Roepstorff A, Rees G, et al.. (2012) Coming to terms quantifying the benefits of linguistic coordination. Psychol. Sci., vol. 23, no. 8, 931–939. 10.1177/0956797612436816 22810169

