# Correction: Learning to Make Collective Decisions: The Impact of Confidence Escalation

**DOI:** 10.1371/annotation/4fcb2fa3-3333-4a4e-9b63-d027f1728912

**Published:** 2014-01-22

**Authors:** Ali Mahmoodi, Dan Bang, Majid Nili Ahmadabadi, Bahador Bahrami

The following information is missing from the funding statement. 

"This work was supported by the Calleva Research Centre for Evolution and Human Sciences (DB)."

The full funding statement is, "AM and MNA are funded by UT and IPM. BB was supported by a postdoctoral fellowship from the British Academy and European Research Council (Grant number: 309865). This work was supported by a StG grant from European Research Council to BB. This work was supported by the Calleva Research Centre for Evolution and Human Sciences (DB). The funders had no role in study design, data collection and analysis, decision to publish, or preparation of the manuscript."

