# Supplementary figures and images for: Learning to Make Collective Decisions: The Impact of Confidence Escalation
Source: PLoS One. 2013 Dec 6;8(12):e81195. doi: 10.1371/journal.pone.0081195 (PMC3855698; doi:10.1371/journal.pone.0081195)

Table S1:


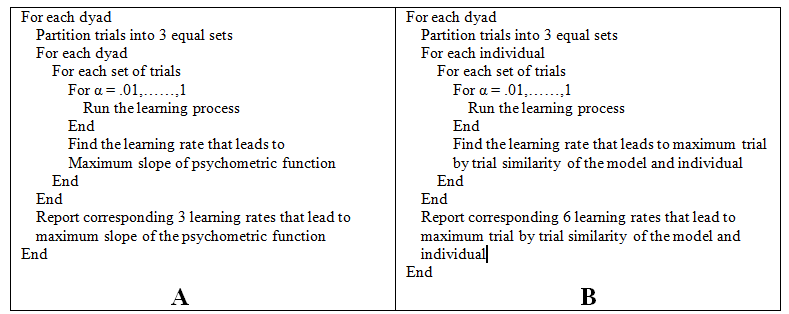

Supplement: Table S1 — Pseudocode for RL algorithm. (A) Maximum accuracy and (B) maximum similarity. In maximum accuracy (maximum similarity) for each dyad (individual) we first transformed the confidence ratings (see Methods) and then ran the learning algorithm with a fixed learning rate for each subset of the experimental data. We searched for the learning rate that maximized the slope (trial by trial similarity of model and individual) over each three subsets of the trials; then for each trial, we assigned decisions to dyads based on the winning learning rate model and finally calculated the overall dyadic slope for each dyad. (DOCX) [file pone.0081195.s001.docx]
